# Antiproliferative Activity of Violaxanthin Isolated from Bioguided Fractionation of *Dunaliella tertiolecta* Extracts

**DOI:** 10.3390/md9050819

**Published:** 2011-05-11

**Authors:** Virginie Pasquet, Perrine Morisset, Said Ihammouine, Amandine Chepied, Lucie Aumailley, Jean-Baptiste Berard, Benoit Serive, Raymond Kaas, Isabelle Lanneluc, Valerie Thiery, Mathieu Lafferriere, Jean-Marie Piot, Thierry Patrice, Jean-Paul Cadoret, Laurent Picot

**Affiliations:** 1 University of La Rochelle, UMR CNRS 6250 LIENSs, F-17042 La Rochelle, France; E-Mails: virginie.pasquet@univ-lr.fr (V.P.); perrinemorisset@hotmail.com (P.M.); said.ihammouine@hotmail.fr (S.I.); amandine.chepied@etudiant.univ-lr.fr (A.C.); lucie.aumailley@etudiant.univ-lr.fr (L.A.); isabelle.lanneluc@univ-lr.fr (I.L.); vthiery@univ-lr.fr (V.T.); mathieu_lafferiere@yahoo.fr (M.L.); jmpiot@univ-lr.fr (J.-M.P.); 2 IFREMER Laboratory PBA, IFREMER Centre Nantes, F-44311 Nantes, France; E-Mails: jean.baptiste.berard@ifremer.fr (J.-B.B.); benoit.serive@ifremer.fr (B.S.); raymond.kaas@ifremer.fr (R.K.); jean.paul.cadoret@ifremer.fr (J.-P.C.); 3 Department LASER, CHU Nantes, F-44093 Nantes, France; E-Mail: thierry.patrice@chu-nantes.fr

**Keywords:** pigments, microalgae, *Dunaliella*, violaxanthin, carotenoid, cancer, apoptosis

## Abstract

*Dunaliella tertiolecta* (*DT*) was chemically investigated to isolate molecules inhibiting cancer cell proliferation and inducing apoptosis *in vitro*. The potency to inhibit cell growth was used for the bio-guided fractionation and isolation of active compounds using chromatographic techniques. The *DT* dichloromethane extract exhibited a strong anti-proliferative activity on MCF-7 and LNCaP cells, and was further fractionated and sub-fractionated by RP-HPLC. High resolution mass spectrometry and spectrophotometric analysis unequivocally identified violaxanthin as the most antiproliferative molecule present in *DT* DCM extract. Violaxanthin purified from *DT* induced MCF-7 dose-dependent growth inhibition in continuous and discontinuous treatments, at concentrations as low as 0.1 μg·mL^−1^ (0.17 μM). Phosphatidylserine exposure, typical of early apoptosis, was observed after 48 h treatment at 8 μg·mL^−1^ (13.3 μM) but no DNA fragmentation, characteristic of late apoptosis steps, could be detected even after 72 h treatment at 40 μg·mL^−1^ (66.7 μM). Taken together, our results demonstrate the strong antiproliferative activity of violaxanthin on one human mammary cancer cell line, and suggest that studying the pharmacology of violaxanthin and pharmacomodulated derivatives on cancer cells may allow potent antiproliferative drugs to be obtained.

## 1. Introduction

Despite significant progress in prevention, diagnosis, and development during the last 25 years, cancer still represents the second cause of mortality in developed countries, after cardiovascular diseases. Discovering new drugs that are more active, more selective, and less toxic, limiting deleterious side effects and tumor multidrug resistance, will obviously be a challenge for the 21st century. The isolation of potent anticancer molecules from the marine environment has generated interest in many groups to purify original compounds, understand their biological activity, and also identify the pharmacological targets of molecules previously known for their ecological function. Extensive screening of marine microalgae has led to the isolation and chemical determination of over 15,000 compounds, including fatty acids, sterols, phenolic compounds, terpenes, enzymes, polysaccharides, alkaloids, toxins and pigments [1]. The pharmacology of most of these molecules remains to be clearly established. Bio-guided fractionation of microalgae extracts, followed by studies on human cells, has demonstrated that many pigments, beyond their ecological function as light harvesting molecules, also act as potent bioactive compounds on cancer cells and may have great potential in the prevention and treatment of cancers [2]. In particular, carotenoids have received increasing attention because of the decreased incidence of cancers associated with their consumption in fruits and vegetables [3,4]. Marine microalgae contain up to 0.2% of carotenoids (w:w dry weight) and may thus be of high interest as functional food to prevent cancer, or as a source of pure carotenoids [5–7]. A large number of studies have demonstrated that purified carotenoids (β,β-carotene, β,α-carotene, lutein, zeaxanthin, lycopene, fucoxanthin, astaxanthin, neoxanthin) exert a direct antiproliferative activity on cancer cells grown *in vitro* and induce their apoptosis [8–15]. The molecular mechanisms ruling this cytotoxicity remain to be clearly established as a large variety of pharmacological effectors regulating cell proliferation, differentiation and apoptosis are affected by carotenoids.

As part of our ongoing activity dedicated to the research and pharmacomodulation of natural anticancer compounds, we screened extracts from various microalgae species, in order to purify and identify antiproliferative molecules. We report here the bioassay-guided isolation of violaxanthin as the major antiproliferative pigment in the dichloromethane extract of the Chlorophyceae *Dunaliella tertiolecta*. Violaxanthin exerted a potent antiproliferative activity on MCF-7 breast cancer cells, and induced biochemical changes typical of early apoptosis.

## 2. Results and Discussion

### 2.1. Antiproliferative Activity of Microalgae Extracts

For each extract, the concentration inhibiting 50% of cell growth (Growth Inhibition 50%; GI_50_) was determined (Table 1).

DCM and EtOH *DT* extracts inhibited MCF-7 growth with equivalent potency and at low concentrations (GI_50_ ≈ 60 μg·mL^−1^). The *DT* DCM extract also inhibited LNCaP growth, with a GI_50_ close to the value determined on MCF-7 (GI_50_ = 60.9 μg·mL^−1^). No extract inhibited MDA-MB-231 growth. The *DT* DCM extract, active both on MCF-7 and LNCaP cells, was selected to purify antiproliferative molecules by fractionation.

### 2.2. RP-HPLC Analysis, Fractionation and Sub-Fractionation of the DT DCM Extract

**Figure 1.**
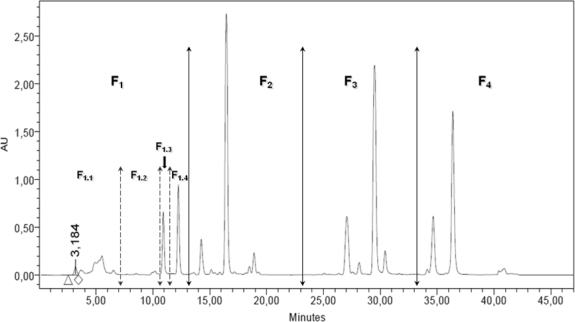
RP-HPLC chromatogram at 435 nm of *Dunaliella tertiolecta* (*DT*) DCM extract. Definition of the four fractions and the four sub-fractions to be collected by semi-preparative RP-HPLC.

Figure 1 presents the *DT* DCM extract chromatogram obtained at 435 nm, with the definition of the fractions and sub-fractions tested on MCF-7.

Identification of the 11 major peaks present on the chromatogram was not performed at this step as we hypothesized that some fractions may not be active on cancer cells. The antiproliferative activity of each fraction was studied on the MCF-7 cell line as it was the most sensitive to the starting DCM extract, and grew faster than LNCaP. Table 2 presents the antiproliferative activity of the four *DT* DCM fractions and the four F1 sub-fractions on MCF-7.

**Figure 2.**
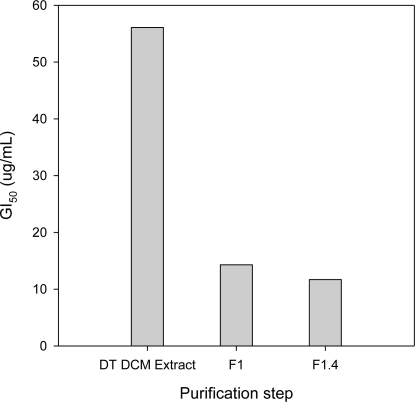
GI_50_ (μg·mL^−1^) of *DT* DCM extract, F_1_ fraction and F_1.4_ sub-fraction on MCF-7.

Fraction 1 (F_1_) was identified as the only active fraction in the *DT* DCM extract, with a GI_50_ = 14.3 μg·mL^−1^. Decrease of the GI_50_ value compared to the DCM extract confirmed that this fraction was concentrated in active molecules (Table 2). The GI_50_ of F_2_, F_3_ and F_4_ were superior to 100 μg·mL^−1^ (Table 2), indicating that they did not contain potent antiproliferative molecules. F_1.2_, F_1.3_ and F_1.4_ strongly inhibited MCF-7 growth, with GI_50_ values of 20.5, 18.9 and 11.7 μg·mL^−1^, respectively (Table 2). The GI_50_ values of these three sub-fractions were in the range of that of the F_1_ fraction, and confirmed that the three sub-fractions contained active molecules (Table 2). The GI_50_ of F_1.1_ was greater than 40 μg·mL^−1^. Figure 2 presents the GI_50_ (μg·mL^−1^) measured on MCF-7 with the starting *DT* DCM extract, the F_1_ fraction and the F_1.4_ subfraction.

The GI_50_ decreased with purification steps, indicating that the antiproliferative activity measured in the initial crude extract was not due to a synergistic action between several molecules in the mixture.

### 2.3. Effect of the F_1.4_ Sub-Fraction on MCF-7 Growth

**Figure 3.**
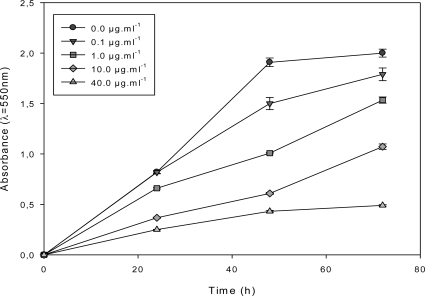
Growth kinetics of MCF-7 continuously treated with the *DT* DCM sub-fraction F_1.4._

The antiproliferative activity of the most active sub-fraction, F_1.4_, was assessed on MCF-7 continuously exposed for 72 h to increasing concentrations in the cell culture medium. F_1.4_ inhibited MCF-7 growth at a concentration as low as 0.1 μg·mL**^−^**^1^ and in a dose-dependent manner from 0.1 to 40 μg·mL^−1^ (Figure 3).

**Figure 4.**
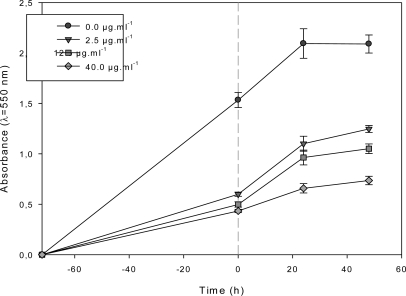
Growth kinetics of MCF-7 during discontinuous exposure to the *DT* DCM sub-fraction F_1.4_ Change to control medium was made at *t*_0_ (gray dots).

A concentration of 40 μg·mL^−1^ was necessary to observe a cytostatic activity on MCF-7 (Figure 3). MCF-7 cells were also exposed for 72 h to various concentrations of F_1.4_ in the cell culture medium, before changing the medium to a fresh control cell culture medium (Figure 4).

At all tested concentrations, the growth rate increased when the culture medium containing F_1.4_ was replaced with fresh culture medium, demonstrating that F_1.4_ exerted a strong antiproliferative effect, without however killing all cells, in the range of the pharmacological concentrations studied.

### 2.4. Characterization of the Antiproliferative Molecule Contained in F_1.4_

**Figure 5.**
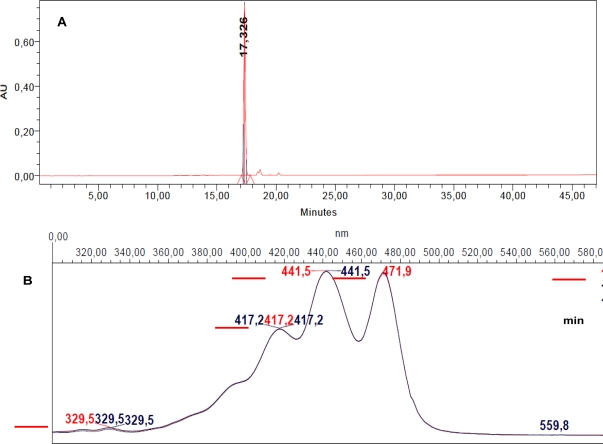
(**A**) RP-HPLC chromatogram of fraction F_1.4_ at 435 nm. F_1.4_ is mainly composed of a single molecule M eluting at *t* = 17.326 min; (**B**) Absorption spectrum of M at *tr* = 17.23, 17.33 and 17.50 min (peak start, peak maximum, peak end).

Analytical RP-HPLC at 435 nm of F_1.4_ demonstrated that 95% of this fraction corresponded to a single molecule M eluting at *t* = 17.326 min (Figure 5).

**Figure 6.**
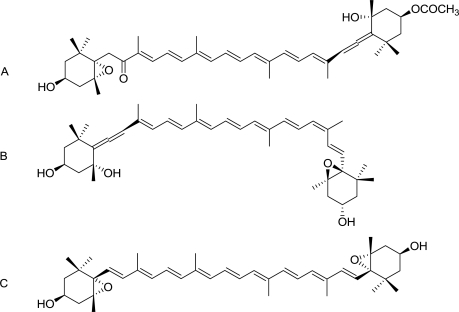
Chemical structures of (**A**) fucoxanthin, (**B**) neoxanthin and (**C**) violaxanthin.

Iterative semi-preparative RP-HPLC allowed the collection of 0.910 mg of F_1.4_ from 1500 mg freeze-dried *DT* cells, indicating that M represented 0.0576% (w:w) of the freeze-dried microalgae content, considering the extraction yield to be 100%. The absorption spectrum of M was characteristic of a carotenoid pigment and presented maximal absorption peaks at 417.2, 441.5 and 471.9 nm, with a band III:II ratio of 96% (Figure 5). These values were compared to data from reference spectra [16] which suggested that M was most probably violaxanthin, as its maximal absorption wavelengths and band III:II ratio were very close to the values measured with standard violaxanthin in ethanol. HRMS analysis unambiguously confirmed that M corresponded to violaxanthin (Table 3; Figure 6C).

### 2.5. Violaxanthin Induces Morphological and Biochemical Changes Characteristic of Early Apoptosis in MCF-7

**Figure 7.**
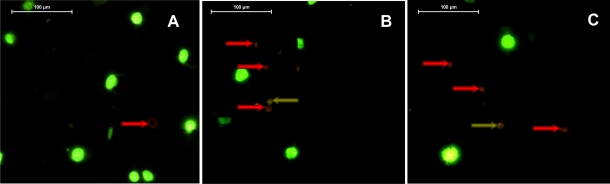
Violaxanthin induces apoptosis of MCF-7 cells. Cells were incubated for 48 h in the presence of 0, 8 or 20 μg·mL^−1^ of violaxanthin (**A**, **B** and **C**, respectively). Fluorochromes: BOBO-1 (green) and Annexin-V-Alexa 568 (red).

Figure 7 shows epifluorescence microphotographs of MCF-7 cells labeled with the DNA marker BOBO-1 (green) and Annexin-V-Alexa 568 (red) after 48 h incubation in the presence of various concentrations of violaxanthin purified from *DT*.

Only a small proportion of control cells (Figure 7A) were identified as apoptotic (red arrows) or early necrotic (bright green spots). Violaxanthin 8 and 20 μg·mL^−1^ (Figures 7B and 7C, respectively) evoked MCF-7 apoptosis as indicated by the important increase in the number of cells binding annexin V without being labeled by BOBO-1. Only a few cells were identified as late necrotic after the violaxanthin treatment (green arrows).

### 2.6. Violaxanthin Does Not Evoke MCF-7 DNA Fragmentation

**Figure 8.**
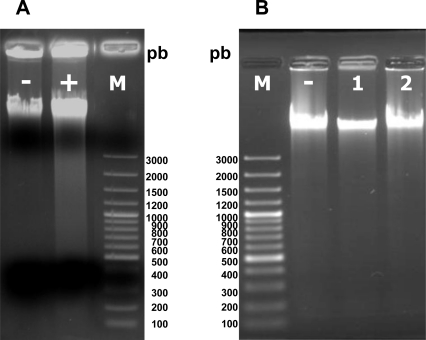
(**A**) Agarose gel electrophoresis of DNA extracted from MCF-7 cells incubated for 72 h in the absence (lane –) or presence of 3,3′-Diindolylmethane 50 μM (DIM, control apoptosis inducer) (lane +); (**B**) Agarose gel electrophoresis of DNA extracted from MCF-7 cells treated with violaxanthin. MCF-7 cells were incubated for 72 h in the absence (lane –) or presence of violaxanthin 10 μg·mL^−1^ (lane 1) and 40 μg·mL^−1^ (lane 2).

Violaxanthin doses evoking phosphatidylserine translocation in MCF-7 cells did not induce DNA fragmentation, even after a 72 h treatment (Figure 8B).

A higher dose of violaxanthin was tested, in the range of rational pharmacological doses (40 μg·mL^−1^) (66.4 μM), but no DNA fragmentation was observed even after 72 h incubation (Figure 8).

## 3. Experimental Section

### 3.1. Microalgae

*Dunaliella tertiolecta* (*DT*) belongs to the Chlorophyceae class (green microalgae). The genus *Dunaliella* exhibit a small ovoid unfrustulated cell (<20 μm), covered of cellulose, xylans, mannans and/or glycoproteins, with two equal flagella inserted at the apexes of the cell. Chlorophyll *a*, chlorophyll *b*, β,β-carotene, neoxanthin, lutein and violaxanthin are the main pigments described in *DT* [17]. *DT* was selected as it does not contain fucoxanthin, which has already been extensively studied for its antiproliferative activity [9–15].

### 3.2. Microalgae Culture, Collection and Storage

Microalgae were grown at IFREMER PBA, Nantes, France. *DT* strain CCMP364 (CCMP, USA) was cultivated in 10 L flasks under continuous illumination at an average light intensity of 180 μmol·m^−2^s^−1^. Growth was performed at 20 °C, in pH unregulated batch culture, in Walne (Conway) medium diluted in 0.22 μm sterile-filtered natural seawater. The *DT* cell suspension was harvested at the end of the exponential growth phase, and cells were separated from culture medium by soft centrifugation (4000 g, 20 min, 10 °C). Cells were frozen at −20 °C, sent to laboratory LIENSs, La Rochelle and freeze-dried at −55 °C and *P* < 1 hPa, on a freeze-dryer equipped with a HetoLyoPro 3000 condenser and Heto cooling trap (Therma Electron Corporation, France).

### 3.3. Successive Extractions in Dichloromethane, Ethanol and Water

In order to extract most microalgal organic molecules, on a wide range of polarity, successive extractions were performed in dichloromethane (DCM), ethanol (EtOH) and ultrapure water. A 1 g sample of freeze-dried microalgae powder was first extracted for 2 h in 100 mL DCM (1% w/v), at room temperature, under continuous shaking and in the dark. The mixture was filtered through a PVDF 0.22 μm membrane, and the DCM extract was evaporated to dryness in dark vials (45 °C, vacuum). The insoluble residue was collected, dried and successively extracted in ethanol and ultrapure water, in the same conditions, except that the water extract was collected by centrifugation (8000 g, 10 min, 4 °C) and filtered through a nitrocellulose membrane. The EtOH extract was evaporated to dryness in dark vials (45 °C, vacuum) and the aqueous extract was freeze-dried.

### 3.4. RP-HPLC Analysis and Fractionation

The RP-HPLC system was composed of a binary pump (Waters, W600), an autosampler (Waters, W717), a thermostated (20 °C) column compartment, and a photodiode-array detector (Waters, W486). Analytical RP-HPLC of the DCM extract was performed on a 20 μL sample injected in a Phenomenex Luna C18 (2) analytical column (250 × 4.6 mm, 10 μm), the mobile phase consisting of a ternary gradient of solvent A (Methanol/water (80/20)); solvent B (Acetonitrile/water (90/10)) and solvent C (ethyl acetate). The gradient flow program was adapted from Jeffrey and collaborators [18], as follows: 0 min, 100% A; 3 min, 100% B; 35 min, 30% B and 70% C; 38 min, 100% C; 41 min, 100% C; 43 min, 100% B; 45 min, 100% A. The flow rate was 1.0 mL·min^−1^ and elution was monitored at 435 nm. Fractionation of the active extract was performed in semi-preparative conditions, using a semi-preparative Phenomenex Luna C18 (2) column (250 × 10 mm, 10 μm) with a flow rate fixed at 5 mL·min^−1^.

### 3.5. Cell Culture

MCF-7 and MDA-MB-231 human mammary carcinoma cell lines, as well as A549 human lung adenocarcinoma cell line (LGC Standards, France) were grown as monolayers, at 37 °C in a 5% CO_2_–95% air humidified atmosphere, in DMEM (Gibco, France) supplemented with 10% heat-inactivated (56 °C, 30 min) FCS (Dutscher, France) to which penicillin 100 U·mL^−1^ and streptomycin 100 μg·mL^−1^ were added. LNCaP human prostatic carcinoma was grown as monolayer in RPMI (devoid of phenol red) supplemented with 10% heat-inactivated (56 °C, 30 min) FCS, penicillin 100 U·mL^−1^ and streptomycin 100 μg·mL^−1^.

### 3.6. Cell Viability Assay

The algal extracts and related fractions were evaporated to dryness and stock solutions were prepared in DMSO before being diluted in the cell culture medium. The final DMSO concentration was lower than 1% and tested as a negative control. Cell viability was studied using the MTT assay.

### 3.7. Detection of Early and Late Steps of Apoptosis

#### 3.7.1. Phosphatidylserines Translocation and DNA Staining

Ten thousand cells were grown for 24 h on epifluorescence live cell array slides (Nunc, Dutscher, France) and treated for 72 h with control culture medium or microalgae extracts diluted in cell culture medium. Phosphatidylserines translocation onto the outer side of the plasma membrane of early apoptotic cells was detected using Annexin-V-Alexa 568 fluorochrome (Roche, France). Since necrotic cells also expose phosphatidylserines because of the loss of membrane integrity, necrotic cells were distinguished from apoptotic cells by BOBO-1 labeling. BOBO-1 is a DNA-binding fluorochrome, excluded from living and apoptotic cells. Cells were incubated for 15 min at 20 °C with the labeling mix solution, and observed using a Leica epifluorescence microscope, equipped with an I3 epifluorescence filter block (blue excitation 450–490 nm) and a numeric camera. Early necrotic cells, having lost their cytoplasmic membrane integrity but with a round shaped nucleus, were labeled by BOBO-1 only and appeared as round green spots. Early apopotic cells, having exposed phosphatidylserines but with no cytoplasmic membrane damages, appeared as red spots. Late necrotic cells exhibited a red and green co-staining and a shrunk nucleus.

#### 3.7.2. DNA Fragmentation

DNA was extracted and purified from cancer cells using a combination of two kits (Macherey-Nagel). In a first step, 10^6^ MCF-7 cells were lysed at 70 °C for 30 min in a lysis buffer (112 μL buffer T1, 25 μL proteinase K and 112 μL buffer B3 from the “Genomic DNA from Tissue” Kit). Then, DNA was purified using 360 μL of BB buffer (“Circulating DNA from Plasma” kit), which allows the purification of high and low molecular weight DNA fragments. DNA samples (5 μg) from control cells, cells treated with violaxanthin or cell treated with a control apoptosis inducer, DIM (3,3′-Diindolylmetane), were separated by electrophoresis on a 1.5% agarose/Tris–borate–EDTA (TBE) gel, stained with ethidium bromide and compared to standards ranging from 100 to 3000 bp (Fermentas) using a UV transilluminator.

### 3.8. High Resolution Mass Spectrometry (HRMS)

Accurate molecular weight of the bioactive molecules contained in *DT* extracts was determined by HRMS at the “Centre Régional de Mesures Physiques de l’Ouest”, University of Rennes 1, France. The mass spectrometer was a Bruker MicrO-Tof-Q 2, equipped with an ESI source, and samples were dissolved in CH_2_Cl_2_:CH_3_OH (90:10).

### 3.9. Chemicals and Standards

All solvents used in this study were HPLC grade. Standard pigments were obtained from Chromadex, France. Ultra-pure water was obtained using a Milli-Q system (Millipore, France).

## 4. Conclusions

Breast cancer is a major cause of mortality worldwide, and the development of new drugs is necessary to reduce mortality and limit tumor resistance to chemotherapy. This study unequivocally demonstrates the strong antiproliferative activity of violaxanthin on MCF-7 human mammary cancer cells grown *in vitro* and suggests that violaxanthin and derivatives obtained by pharmacomodulation should be studied as possible new drugs to cure breast cancer. Violaxanthin was already identified as possibly involved in the strong antiproliferative and pro-apoptotic activity of *Chlorella ellipsoidea* extracts on HCT116 human colon cancer cells [19]. Further studies should be undertaken to define the pharmacological mechanisms involved in its antiproliferative activity in human cancer cells. In this study, violaxanthin only represented 0.0576% (w:w) of the total freeze-dried *DT* content, with a theoretical extraction yield of 100%. Thus, it seems unrealistic to consider that violaxanthin could be purified from *DT* for therapeutic applications, even if physiological studies in *Dunaliella* or *Chlorella* may be carried out to increase production yields. Moreover, there is no clear evidence that epoxycarotenoids, despite their abundance in dietary fruits and vegetables, are absorbed *per os* by humans [20]. Recent studies indicate that epoxycarotenoids are metabolized before being absorbed *per os* in mice [21], suggesting that consumption of marine microalgae as functional food to obtain epoxycarotenoids for a putative cancer prevention or treatment is most probably less beneficial. It is, however, interesting to note that the oral administration of high doses of fucoxanthin in mice does not evoke acute or chronic toxicity, except the risk of hypercholesterolemia [22]. Additional studies will be necessary to get a clear understanding of epoxycarotenoids pharmacology and to consider their possible use to inhibit cancer cells growth *in vivo*.

## Figures and Tables

**Table 1.** GI_50_ (μg·mL^−1^) of *Dunaliella tertiolecta* extracts on four cancer cell lines. EtOH: ethanol; DCM: dichloromethane; ≫ means GI_50_ > 100 μg·mL^−1^.

**Extraction solvent**	**Cell line**			
	**MCF-7**	**MDA-MB-231**	**A549**	**LNCaP**
Water	≫	≫	≫	≫
EtOH	61.5	≫	≫	≫
DCM	56.1	≫	≫	60.9

**Table 2.** GI_50_ (μg·mL^−1^) of *DT* DCM fractions and sub-fractions on the MCF-7 cell line. ≫ means GI_50_ > 100 μg·mL^−1^; > means GI_50_ > 40 μg·mL^−1^.

***DT* DCM fractions**	**F_1_**	**F_2_**	**F_3_**	**F_4_**
GI_50_ (μg·mL^−1^)	14.3	≫	≫	≫
***DT* DCM sub-fractions**	**F_1.1_**	**F_1.2_**	**F_1.3_**	**F_1.4_**
GI_50_ (μg·mL^−1^)	>	20.5	18.9	11.7
SEM GI_50_ (μg·mL^−1^)		2.2	8.85	0.2

**Table 3.** High resolution mass spectrometry (HRMS) identification of F_1.4_.

**Lead compound F_1.4_**	
Molecular formula	[M + Na]^+^ (C_40_H_56_O_4_Na)
Theoretical molecular weight	623.40763
*z*	1
Theoretical *m/z* value	623.40708
Experimental *m/z* value [M + Na]	623.4068 (0 ppm)

## References

[1] Kornprobst J-M (2005). Substances Naturelles D’origine Marine: Chimiodiversité, Pharmacodiversité, Biotechnologies.

[2] Folmer F, Jaspars M, Dicato M, Diederich M (2010). Photosynthetic marine organisms as a source of anticancer compounds. Phytochem Rev.

[3] Nishino H, Murakoshi M, Tokuda H, Satomi Y (2009). Cancer prevention by carotenoids. Arch Biochem Biophys.

[4] Nishino H, Tokuda H, Murakoshi M, Satomi Y, Masuda M, Onozuka M, Yamaguchi S, Takayasu J, Tsuruta J, Okuda M (2000). Cancer prevention by natural carotenoids. BioFactors.

[5] Del Campo JA, Moreno J, Rodríguez H, Vargas MA, Rivas J, Guerrero MG (2000). Carotenoid content of chlorophycean microalgae: Factors determining lutein accumulation in *Muriellopsis* sp. (Chlorophyta). J Biotechnol.

[6] Dufossé L, Galaup P, Yaron A, Arad SM, Blanc P, Chidambara Murthy KN, Ravishankar GA (2005). Microorganisms and microalgae as sources of pigments for food use: A scientific oddity or an industrial reality. Trends Food Sci Technol.

[7] Milledge JJ (2010). Commercial application of microalgae other than as biofuels: A brief review. Rev Environ Sci Biotechnol.

[8] Cui Y, Lu Z, Bai L, Shi Z, Zhao WE, Zhao B (2007). β-Carotene induces apoptosis and up-regulates peroxisome proliferator-activated receptor γ expression and reactive oxygen species production in MCF-7 cancer cells. Eur J Cancer.

[9] Kotake-Nara E, Asai A, Nagao A (2005). Neoxanthin and fucoxanthin induce apoptosis in PC-3 human prostate cancer cells. Cancer Lett.

[10] Das SK, Hashimoto T, Kanazawa K (2008). Growth inhibition of human hepatic carcinoma HepG2 cells by fucoxanthin is associated with down-regulation of cyclin D. Biochim Biophys Acta Gen Subj.

[11] Das SK, Hashimoto T, Shimizu K, Yoshida T, Sakai T, Sowa Y, Komoto A, Kanazawa K (2005). Fucoxanthin induces cell cycle arrest at G0/G1 phase in human colon carcinoma cells through up-regulation of p21WAF1/Cip1. Biochim Biophys Acta Gen Subj.

[12] Hosokawa M, Kudo M, Maeda H, Kohno H, Tanaka T, Miyashita K (2004). Fucoxanthin induces apoptosis and enhances the antiproliferative effect of the PPARγ ligand, troglitazone, on colon cancer cells. Biochim Biophys Acta Gen Subj.

[13] Hosokawa M, Wanezaki S, Miyauchi K, Kurihara H, Kohno H, Kawabata J, Odashima S, Takahashi K (1999). Apoptosis-inducing effect of fucoxanthin on human leukemia cell line HL-60. Food Sci Technol Res.

[14] Moreau D, Tomasoni C, Jacquot C, Kaas R, Le Guedes R, Cadoret JP, Muller-Feuga A, Kontiza I, Vagias C, Roussis V (2006). Cultivated microalgae and the carotenoid fucoxanthin from *Odontella aurita* as potent anti-proliferative agents in bronchopulmonary and epithelial cell lines. Environ Toxicol Pharm.

[15] Nakazawa Y, Sashima T, Hosokawa M, Miyashita K (2009). Comparative evaluation of growth inhibitory effect of stereoisomers of fucoxanthin in human cancer cell lines. J Funct Foods.

[16] Jeffrey SW, Mantoura RFC, Wright SW (1997). Phytoplankton Pigments in Oceanography.

[17] Jeffrey SW, Mantoura RFC, Wright SW (1997). Phytoplankton Pigments in Oceanography.

[18] Jeffrey SW, Mantoura RFC, Wright SW (1997). Phytoplankton Pigments in Oceanography.

[19] Cha KH, Koo SY, Lee D (2008). Antiproliferative effects of carotenoids extracted from *Chlorella ellipsoidea* and *Chlorella vulgaris* on human colon cancer cells. J Agric Food Chem.

[20] Barua AB (1999). Intestinal absorption of epoxy-β-carotenes by humans. Biochem J.

[21] Hashimoto T, Ozaki Y, Taminato M, Das S, Mizuno M, Yoshimura K, Maoka T, Kanazawa K (2009). The distribution and accumulation of fucoxanthin and its metabolites after oral administration in mice. Br J Nutr.

[22] Beppu F, Niwano Y, Sato E, Kohno M, Tsukui T, Hosokawa M, Miyashita K (2009). *In vitro* and *in vivo* evaluation of mutagenicity of fucoxanthin (FX) and its metabolite fucoxanthinol (FXOH). J Toxicol Sci.

